# Effect of exclusive breastfeeding on selected adverse health and nutritional outcomes: a nationally representative study

**DOI:** 10.1186/s12889-017-4913-4

**Published:** 2017-11-21

**Authors:** Md. Nuruzzaman Khan, M. Mofizul Islam

**Affiliations:** 1 Department of Population Sciences, Jatiya Kabi Kazi Nazrul Islam University, Mymensingh, 2220 Bangladesh; 2 Department of Public Health, La Trobe University, Melbourne, Australia; 3 Department of Health Services Research and Policy, Australian National University, Canberra, Australia

**Keywords:** Exclusive breastfeeding; child health, Under-nutrition, World Health Organization recommendation, Bangladesh

## Abstract

**Background:**

Despite growing evidence in support of exclusive breastfeeding (EBF) among infants in the first 6 months of birth, the debate over the optimal duration of EBF continues. This study examines the effect of termination of EBF during the first 2, 4 and 6 months of birth on a set of adverse health and nutritional outcomes of infants.

**Methods:**

Three waves of Bangladesh Demographic and Health Survey data were analysed using multivariate regression. The adverse health outcomes were: an episode of diarrhea, fever or acute respiratory infection (ARI) during the 2 weeks prior to the survey. Nutritional outcomes were assessed by stunting (height-for-age), wasting (weight-for-height) and underweight (weight-for-age). Population attributable fraction was calculated to estimate percentages of these six outcomes that could have been prevented by supplying EBF.

**Results:**

Fifty-six percent of infants were exclusively breastfed during the first 6 months. Lack of EBF increased the odds of diarrhea, fever and ARI. Among the babies aged 6 months or less 27.37% of diarrhea, 13.24% of fever and 8.94% of ARI could have been prevented if EBF was not discontinued. If EBF was terminated during 0–2 months, 2–4 months the odds of becoming underweight were 2.16 and 2.01 times higher, respectively, than babies for whom EBF was not terminated.

**Conclusion:**

Children who are not offered EBF up to 6 months of their birth may suffer from a range of infectious diseases and under-nutrition. Health promotion and other public health interventions should be enhanced to encourage EBF at least up to six-month of birth.

**Trail registration:**

Data of this study were collected following the guidelines of ICF International and Bangladesh Medical Research Council. The registration number of data collection is 132,989.0.000 and the data-request was registered on September 11, 2016.

## Background

There is an increasing evidence that exclusive breastfeeding (EBF) up to six-month of age has profound biological effects and important consequences on health and nutritional outcomes of children [1, 2]. The immunological properties of breast milk contribute to ensuring adequate nutritional status, proper growth and develop morbidity prevention capacity in child body [3, 4]. In addition, EBF substantially reduces the risk of morbidity and mortality from infectious diseases by eliminating the chance of contamination from formula milk or other fluids and foods [3].

Although it is now well accepted that EBF is enormously important to infants’ health and nutritional status, the most appropriate age for introducing other food is still debated. Before 2001, the World Health Organization (WHO) recommended introducing solid food at 4–6 months. In 2001 the recommendation was changed to 6 months [5]. This change was, in fact, driven by new evidence about benefits of longer-term EBF against infectious diseases and adequacy of breast milk to meet infants’ nutritional requirements of beyond 4 months of age [6]. However, sometimes the evidence has been questioned [7], and some experts have promoted a less stringent recommendation that is more akin to the pre-2001 WHO policy [8, 9]. Part of this controversy is caused by findings of some individual studies [10]. The debate over the optimal duration of EBF is centered mainly on the choice between the known protective effect of EBF against infectious diseases and the insufficiency of breast milk alone to satisfy babies’ nutritional requirements (known as weanling’s dilemma).

Using nationally representative Bangladesh Demographic and Health Survey (BDHS) data, we examined the effect of termination of EBF during the first 2, 4 and 6 months of birth on a set of adverse health and nutritional outcomes of infants. We also examined the population attributable fraction (PAF) to estimate the percentages of specific health and nutritional outcomes that could have been prevented if EBF was not discontinued during the first 6 months of birth.

## Methods

### Data sources

From three waves of BDHS conducted in 2007, 2010 and 2014 we pooled data of 1918 babies less than 6 months of age. Details of the survey questionnaire and data collection procedure were published elsewhere [11–14]. Briefly, the BDHS used two-stage stratified cluster sampling approach. In the first stage, enumeration areas were selected with probability proportional to their size. In the second stage, a systematic sample of 30 households on average was selected from each sampling unit to provide statistically reliable estimates of key demographic and health variables for the country as a whole, for urban and rural areas separately, and for each of the seven divisions of Bangladesh. Two different survey instruments were used for each round of survey: household schedule and individual questionnaire for ever-married women of reproductive age (15–49 years old). Information regarding socio-demographic status of households and members were collected by household schedule. The individual questionnaire was used to collect information on women’s reproductive history, their health and nutritional status, antenatal care, delivery and postnatal care, breastfeeding and infant feeding practices, immunization, child health and nutrition. Overall, rural population constituted 67% (1292/1918) and urban population constituted 33% (626/1918) of the sample. In the combined dataset (*n* = 1918) the contribution of three waves of survey were 27%, 41% and 32%, respectively.

### Outcome variables

A set of adverse health and nutritional outcomes among children were the outcome variables. The adverse health outcomes were an episode of diarrhea, fever or ARI during the 2 weeks prior to the survey. The nutritional outcomes were assessed by stunting (height-for-age), wasting (weight-for-height) and underweight (weight-for-age) classified by WHO growth standard. Adverse health outcomes were ascertained from responses to the following questions: (a) has the baby had any diarrhea in the last 2 weeks? (b) has the baby been ill with a fever at any time during the last 2 weeks? (c) did the baby breathe faster than usual with short, rapid breaths or have difficulty breathing when the baby had an illness with a cough? and (d) was the fast or difficult breathing due to a problem in the chest or to a blocked or runny nose? Responses were recorded “Yes” vs “No”. A child was considered as having experienced ARI if child’s mother reported that during the last 2 weeks preceding the survey the child had a cough along with short, rapid breathing accompanied by a fever. Three different anthropometric measurements (weight, height, age) were taken during the survey to derive the nutritional outcomes.

### Exposure variable

Status of EBF from birth to 6 months of age was the key exposure variable. During the survey, respondents were asked (i) whether the baby was still being breastfed; (ii) the duration of breastfeeding; and (iii) if other foods were given during the last 24 h. Finally, to examine the effect of the duration of EBF, we categorized the sample in three subgroups based on EBF cessation time (0–2 months, 2–4 months, and 4–6 months).

### Statistical analysis

We used mean and its 95% confidence interval (95% CI) to describe selected demographic information, health and nutritional outcomes of babies and their mothers. The association between EBF-termination and selected health and nutritional outcomes was investigated by both unadjusted and adjusted multivariate logistic regression. Covariates that could be consistently measured across the three surveys and that were found important in the literature were included in adjusted multivariate models [15–18]. They were maternal age at birth (≤19, 20–34, ≥35), place of residence (urban, rural), region of residence (seven divisions: Barisal, Dhaka, Chittagong, Khulna, Rajshahi, Rangpur, Sylhet), number of antenatal visits (no visit, 1–4 visit, >4 visit), wealth quintile (poorest, poorer middle, richer, richest), maternal education (none, primary, secondary, higher education), method of delivery (cesarean, vaginal delivery), birth weight (normal, low birth weight) and the survey years. We included ‘household use of solid fuel’ in the adjusted models for ARI, as some previous studies found this variable significantly associated with ARI [19, 20]. All analyses were adjusted for the complex survey design with necessary weight for sampling. Finally, the population attributable fraction (PAF) for each of the outcome variables was calculated using the following formula: $\frac{\mathrm{Prevalence}\  \mathrm{of}\  \mathrm{exposure}\ \left(\mathrm{OR}-1\right)}{1+\mathrm{prevalence}\  \mathrm{of}\  \mathrm{exposure}\ \left(\mathrm{OR}-1\right)}$; where OR is odds ratio for exposure (in this context, not-EBF to EBF) [21, 22].

## Results

A total of 1918 babies aged 6 months or below were included in our study. Around 56% were exclusively breastfed up to the first 6 months of birth. The mean age of mothers at the time of delivery was 24 years (SD ±5.59) and babies was 2.65 years (SD ±1.65). There were almost equal number of male and female babies. Majority of mothers (65%) were formally educated (at least primary) and did not have any formal occupation (85%). Around 42% of women were from poor wealth quintile. Mean number of antenatal visit was 2.59 times during pregnancy. Table 1 shows the socio-demographic characteristics of mothers and their babies.

**Table 1:** Breastfeeding, infant and maternal characteristics

Parameter	Mean/Prevalence (95% CI)
Perinatal and Infant characteristics	
Exclusively breastfed %	56.4 (53.2–59.5))
Child age in months, mean	3.23 (3.16–3.32)
Female child %	48.5 (47.1–49.9)
Delivery by cesarean section %	18.2 (17.0–19.5)
Low birth weight %	19.4 (18.0–20.8)
Maternal and Household characteristics	
Age at delivery, mean	23.6 (23.5–23.7)
No formal occupation %	84.7 (83.4–85.9)
Formal services %	15.3 (14.1–16.6)
Institutional education (primary or above) %	64.8 (63.1–66.4)
Household poor wealth quintile %	41.6 (39.743.6)
Household rich wealth quintile %	38.5 (36.3–40.6)
Antenatal care received in number, mean	2.59 (2.53–2.64)
Birth interval in months, mean	56.4 (55.5–57.3)
Prevalence of adverse health and nutritional outcome	
Diarrhea in the two weeks prior to the survey %	8.3 (7.5–9.3)
Fever in the two weeks prior to the survey %	42.2 (40.7.9–43.6)
Acute respiratory infection in the two weeks prior to the survey %	18.5 (17.5–19.5)
At least one of the above three health outcomes during two weeks prior to the survey %	48.9 (47.5–50.2)
Stunting%	18.1 (17.2–19.3)
Wasting %	17.3 (16.0–18.5)
Underweight %	28.8 (27.5–30.2)
At least one of the above three nutritional outcomes %	49.6 (48.2–51.1)

The prevalence of diarrhea was 8.3%. Around 42% of babies suffered from fever during the 2 weeks prior to the surveys. The prevalence of ARI was around 18.5%. Overall, almost half of the babies (48.9%) suffered from at least one of these three diseases during the 2 weeks preceding the surveys. The prevalence of stunting and wasting was 18.1 and 17.3%, respectively. Almost 30% of the children were underweight during the survey. Overall, 50% of children were found to have one of the three types of under-nutrition (i.e. stunting, wasting and underweight).

Table 2 shows unadjusted and adjusted odds ratios (ORs) and their corresponding confidence intervals for diarrhea, fever and ARI. EBF has a significant effect on each of these three diseases before and after adjusting for possible confounding factors. However, the effect gradually decreases with the increase in duration of EBF. If EBF was terminated during 0–2 months, 2–4 months and 4–6 months the odds for babies in getting diarrhea were 4.94 times (95% CI, 3.17–10.23), 3.07 times (95% CI, 2.11–5.03) and 2.30 times (95% CI, 1.89–3.20) higher, respectively, than babies for whom EBF was not terminated. Babies were 2.18 times (95% CI, 1.56–3.04), 1.53 times (95% CI, 1.37–2.10) and 1.23 times (95% CI, 1.06–1.63) more likely to get fever if EBF was terminated during 0–2, 2–4 and 4–6 months, respectively, than the babies for whom EBF was not terminated. Similar results were found for ARI (Table 2). Overall, the ORs of getting at least one of these three diseases was 2.50, 1.64 and 1.42 if EBF was terminated during 0–2, 2–4 and 4–6 months, respectively, in reference to the babies for whom EBF was not terminated.

**Table 2:** Association between breastfeeding and selected adverse health outcomes

Outcome variable	EBF terminated between 0 and 2 months (*n* = 567) ^a^		EBF terminated between 2 and 4 months (*n* = 1223) ^a^		EBF terminated between 4 and 6 months (*n* = 1918) ^a^		PAF %
	Crude OR (95% CI)	Adjusted OR (95% CI)	Crude OR (95% CI)	Adjusted OR (95% CI)	Crude OR (95% CI)	Adjusted OR (95% CI)	
Diarrhea	5.80 (3.19–11.63)	4.94 (3.17–10.23)	3.13 (2.04–4.87)	3.07 (2.11–5.03)	2.34 (1.89–3.11)	2.30 (1.89–3.20)	27.37
Fever	2.24 (2.00–2.98)	2.18 (1.56–3.04)	1.54 (1.35–1.78)	1.53 (1.37–2.10)	1.25 (1.07–1.48)	1.23 (1.06–1.63)	13.24
ARI	2.43 (1.98–3.45)	2.38 (1.27–3.26)	1.42 (1.13–1.75)	1.40 (1.10–1.76)	1.25 (1.03–1.48)	1.19 (1.04–1.57)	8.94
At least one of the above three diseases	2.56 (2.12–3.38)	2.50 (2.10–3.32)	1.63 (1.40–1.95)	1.64 (1.30–1.98)	1.45 (1.22–1.79)	1.42 (1.20–1.76)	10.52

^a^Reference group: EBF not terminated up to six months

Note: n: total sample size, *OR* odds ratio, *CI* confidence interval, *PAF* population-attributable fraction (if EBF was not discontinued during first six months)

Table 3 shows unadjusted and adjusted ORs and their corresponding confidence intervals for stunting, wasting and underweight. EBF has a significant effect against underweight up to 4 months and an insignificant effect thereafter. If EBF was terminated during 0–2 months, 2–4 months the odds of babies of becoming underweight were 2.16 times and 2.01 times higher, respectively, than the babies for whom EBF was not terminated. Termination of EBF at three durations (i.e. 0–2 months, 2–4 months and 4–6 months) does not have any effect on stunting or wasting. The ORs of getting any of the three forms of under-nutrition was 1.54 (CI: 1.23–1.90) and 1.18 (CI: 1.00–1.47) if EBF was terminated during 2–4 months and 4–6 months respectively, in reference to the babies for whom EBF was not terminated.

**Table 3:** Association between breastfeeding and selected nutritional outcomes

Outcome variable	EBF terminated between 0 and 2 months (*n* = 728) ^a^		EBF terminated between 2 and 4 months (*n* = 1648) ^a^		EBF terminated between 4 and 6 months (*n* = 2590) ^a^		PAF %
	Crude OR (95% CI)	Adjusted OR (95% CI)	Crude OR (95% CI)	Adjusted OR (95% CI)	Crude OR (95% CI)	Adjusted OR (95% CI)	
Stunting	1.39 (0.75–2.57)	1.39 (0.71–2.67)	1.18 (0.83–1.69)	1.16 (0.80–1.68)	1.25 (0.97–1.61)	1.18 (0.91–1.54)	2.21
Wasting	0.83 (0.46–1.49)	0.85 (0.45–1.59)	1.26 (0.91–1.75)	1.29 (0.92–1.80)	1.06 (0.83–1.37)	1.07 (0.82–1.39)	0.56
Underweight	2.25 (1.30–3.91)	2.16 (1.19–3.91)	2.17 (1.59–2.97)	2.01 (1.57–3.08)	1.64 (0.80–1.45)	1.76 (0.74–1.49)	5.90
At least one of the above three nutritional outcomes	1.64 (1.18–2.44)	1.43 (0.89–2.14)	1.57 (1.28–2.10)	1.54 (1.23–1.90)	1.23 (1.04–1.44)	1.18 (1.00–1.47)	6.70

^a^Reference group: EBF not terminated up to six months

Note: *n*: total sample size, *OR* odds ratio, *CI* confidence interval, *PAF* population-attributable fraction (if EBF was not discontinued during first six months)

PAFs also show there are protective effects of EBF on adverse health and nutritional outcomes. This protective effect was strongest for diarrhea (27.37%), followed by fever (13.24%) and ARI (8.94%). Overall, around 10% of the reported illness attributable to these three diseases could be prevented if EBF was not discontinued during the first 6 months (Table 2). There were slight protective effects of EBF on under-nutrition; around 6% underweight, 2.21% stunting and 0.56% wasting could be prevented by ensuring EBF during the first 6 months. Overall, around 7% of stunting, wasting or underweight in Bangladesh could have been prevented by ensuring EBF up to 6 months of birth.

## Discussion

Worldwide substantial adverse health and nutritional outcomes are attributed to lack of EBF [23, 24]. Results of this study suggest a protective effect of EBF against diarrhea, ARI and fever. EBF up to 6 months did not seem to cause under-nutrition, rather termination of EBF at 2–4 months were found to be associated with underweight. These findings of EBF with three nutritional outcomes, in fact, suggests that the concern around insufficiency of EBF to fulfill the nutritional requirement is not supported. Therefore, together, our findings suggest EBF up to 6 months is beneficial in protecting babies from infections and sufficient to fulfill the nutritional requirement. Overall, our findings are largely consistent with the recommendation of WHO for EBF during the first 6 months.

Worldwide, diarrhea, fever, ARI and under-nutrition remain the major killers of children under 5 years old (often known as under-five death). Of 10.4 million under-five deaths from preventable causes in developing countries each year, over 50% die due to under-nutrition [25] and 29% due to ARI and diarrhea [26]. These adverse health and nutritional outcomes cause frequent hospitalization, improper physical growth and create a momentum leading to the further morbidity, and frequent school missing [27]. EBF up to 6 months of birth is a feasible and healthy preventive measure against adverse health and nutritional outcomes. In our dataset the overall prevalence of EBF during the first 6 months of birth was 56%, which was 17% higher than the average prevalence of EBF (39%) in developing countries [28]. Clearly, this higher prevalence highlights a public health achievement for Bangladesh. This, however, should not be a reason for complacency, as around 46% of children experience some episodes of diarrhea or ARI during the first year of life and one third of total child death burden is attributed to diarrhea [15]. Therefore, the importance of EBF up to the 6 months of birth is crucial for Bangladesh, as the general hygiene practices and overall sanitation are inadequate.

In our analysis we found EBF up to 6 months could have prevented around 27.37% of reported diarrhea and 8.94% of the ARI cases. These findings are consistent to that reported in the previous literature [16, 17, 29]. Factors associated with the occurrence of diarrhea and ARI are broad – some of them are modifiable [3, 30–32]. The mechanisms through which breastfeeding have protective effects on infectious diseases are multiple. Firstly, human milk has specific immunologic properties that protect the infants from infection [33]. Secondly, the array of antimicrobial, anti-inflammatory, immunomodulatory protection, and bioactive molecules and compounds of breast milk create protections against infection [34]. Thirdly, breast milk promotes mucosal maturation, stimulating neonatal immune systems; limits exposure to the germs from foreign dietary antigens [35, 36].

We found a gradual decrease in the ORs of three durations of EBF (or EBF termination) on diarrhea, fever and ARI. This observation is interesting and important. A reasonable explanation of this gradual duration effect is that babies’ immunity is low soon after their birth. With the help of EBF and natural immunity gradually the body develops prevention capacity. In addition, children without EBF receive foods and drinks which could be contaminated [17, 22], and/or difficult to digest [37]. Particularly in developing countries the lack of hygiene and related knowledge increase this contamination, which is the main reason for high infection rate among children of lower socio-economic background [38]. These factors, together, may explain the gradual protective effects of EBF against infections.

Our findings about little or no significant association of EBF with stunting, wasting and underweight are largely consistent to that from the previous studies conducted in developing countries [39, 40]. The WHO estimates that inappropriate feeding of infant is responsible for one-third of the cases of malnutrition worldwide [41]. Complementary foods are necessary when breast milk alone is no longer able to satisfy the nutritional requirement of the growing infant. Too early or too late introduction may adversely affect the child nutritional status. Particularly in developing countries, an introduction of complementary food too early may cause frequent occurrence of microbial contamination and increase the risk of diarrhea and ARI [39]. Thus the weanling’s dilemma and the risk of morbidity and mortality associated with early introduction of complementary foods are concerns primarily in developing countries. This dilemma divided the public health experts and there are disagreements about the right time of introducing complementary food. Our study findings support EBF during the entire first 6 months, as this protects babies against infections, and that it does not cause insufficient nutritional intake, rather termination of EBF during the first 4 months could cause underweight.

It should be noted here that our study is based on population level data. Thus while our overall findings are supportive of EBF up to 6 months, there may be individual cases or settings where early introduction of complementary foods may deem necessary. For instance, in some developing country settings poverty compels mothers to work outside and far away from home that may necessitate to introduce other foods [42]. In a study in Myanmar, lack of financial resources was also cited as a reason for poor maternal nutrition, leading to reduced breast milk production [42].

The main strength of our study is a population level representative data of recent years and a relatively large sample size. Also exposure and outcome data were collected at the same time by imposing 2 weeks recall period, which is likely to reduce bias [18]. We also adjusted our results for a wide range of socio-demographic and health-related confounders. Moreover, unlike previous literature we provided independent statistics for fever alone. Our study has also some limitations. Our data were cross-sectional, therefore, the relationship is correlational only rather than causal. Secondly, it is possible that the temporal sequence of the early signs of infection and termination of breastfeeding were not adequately appreciated by a subgroup of mothers; infection might have been blamed for the termination of breastfeeding, rather than the reverse (i.e. reverse causality). Data collection within a short duration restricted our scope to take seasonal variation into consideration, which was found important for child morbidities [43, 44]. Also this study does not intend to assess the weanling’s dilemma and the adequacy of nutrition from EBF. Additionally, our data were based on participants’ self-report with no scope of validation by the interviewers. However, an evaluation of Demographic and Health Survey data found that the exposure and outcome variables were reasonably well reported [45].

## Conclusion

Lack of EBF up to 6 months of birth has adverse consequences on the health and nutritional status of children. A substantial proportion infectious disease and under-nutrition could be prevented if EBF was ensured up to 6 months after birth. Our findings were consistent to the WHO recommendation regarding the public health benefit of EBF in the first 6 months of birth. We recommend taking further initiatives to promote EBF in general, and tailoring programs to those women who are not practicing EBF, in particular.

## Abbreviations

aOR: Adjusted Odds Ratio
ARI: Acute Respiratory Infection
BDHS: Bangladesh Demographic and Health Survey
EBF: Exclusive breastfeeding
OR: Odds Ratio
PAF: Population attributable fraction
WHO: World Health Organization

## References

[1] Horta BL, Victora CG. Short-term effects of breastfeeding: A systematic review on the benefits of breastfeeding on diarrhoea and pneumonia mortality. Geneva: The World Health Organization; 2013.

[2] Black RE, Victora CG, Walker SP, Bhutta ZA, Christian P, De Onis M, Ezzati M, Grantham-McGregor S, Katz J, Martorell R (2013). Maternal and child undernutrition and overweight in low-income and middle-income countries. Lancet.

[3] Lamberti LM, Walker CLF, Noiman A, Victora C, Black RE (2011). Breastfeeding and the risk for diarrhea morbidity and mortality. BMC Public Health.

[4] Field CJ (2005). The immunological components of human milk and their effect on immune development in Infants^1, 2^. J Nutr.

[5] WHO (2001). The optimal duration of exclusive breastfeeding: report of an expert consultation.

[6] Kramer M, Kakuma R (2002). Optimal duration of exclusive breastfeeding (review). Cochrane Database Syst Rev.

[7] Fewtrell M, Wilson DC, Booth I, Lucas A (2011). Six months of exclusive breast feeding: how good is the evidence?. BMJ.

[8] Agostoni C, Decsi T, Fewtrell M, Goulet O, Kolacek S, Koletzko B, Michaelsen KF, Moreno L, Puntis J, Rigo J (2008). Complementary feeding: a commentary by the ESPGHAN committee on nutrition. J Pediatr Gastroenterol Nutr.

[9] British Dietetic Association. Complementary feeding: introduction of solid food to an infants diet. Policy statement Birmingham. United Kingdom: The British Dietetic Association; 2013.

[10] Reilly JJ, Wells JC (2006). Duration of exclusive breast-feeding: introduction of complementary feeding may be necessary before 6 months of age-response. Brit J Nutr.

[11] Mitra S, Ali M, Islam S, Cross A, Saha T. Bangladesh demographic and health survey, 2007. Dhaka: The National Institute of Population Research and Training, NIPORT, Mitra and Associates and ORC Macro International Inc; 2008.

[12] Mitra S, Ali M, Islam S, Cross A, Saha T. Bangladesh demographic and health survey, 2011. Dhaka: The National Institute of Population Research and Training, NIPORT, Mitra and Associates and ORC Macro International Inc; 2012.

[13] Mitra S, Ali M, Islam S, Cross A, Saha T. Bangladesh demographic and health survey, 2014. Dhaka: The National Institute of Population Research and Training, NIPORT, Mitra and Associates and ORC Macro International Inc; 2015.

[14] Chowdhury MAB, Uddin MJ, Khan HMR, Haque R (2015). Type 2 diabetes and its correlates among adults in Bangladesh: a population based study. BMC Public Health.

[15] Piechulek H, Al-Sabbir A, Mendoza-Aldana J (2003). Diarrhea and ARI in rural areas of Bangladesh. Southeast Asian J Trop Med Public Health.

[16] Cushing AH, Samet JM, Lambert WE, Skipper BJ, Hunt WC, Young SA, McLaren LC (1998). Breastfeeding reduces risk of respiratory illness in infants. Am J Epidemiol.

[17] Arifeen S, Black RE, Antelman G, Baqui A, Caulfield L, Becker S (2001). Exclusive breastfeeding reduces acute respiratory infection and diarrhea deaths among infants in Dhaka slums. Pediatrics.

[18] Kamal MM, Hasan MM, Davey R (2015). Determinants of childhood morbidity in Bangladesh: evidence from the demographic and health survey 2011. BMJ Open.

[19] Khan MN, B.Nurs CZ, Islam MM, Islam MR, Rahman MM (2017):Household air pollution from cooking and the risk of adverse health and birth outcomes in Bangladesh: A nation wide population based study. Environ Health; 2017; 16(57). 10.1186/s12940-017-0272-y PMC5470285 28610581

[20] Ganguly E, Sharma PK, Bunker CH (2016). Burden of acute infections (except respiratory and diarrheal) and its risk factors among under-five children in India: a systematic review and meta-analysis. Indian J Child Health.

[21] Levin ML (1952). The occurrence of lung cancer in man. Acta-Unio Internationalis Contra Cancrum.

[22] Quigley MA, Kelly YJ, Sacker A (2007). Breastfeeding and hospitalization for diarrheal and respiratory infection in the United Kingdom millennium cohort study. Pediatrics.

[23] Kramer MS, Kakuma R. Optimal duration of exclusive breastfeeding. Geneva: The World Health Organization; 2002.

[24] Jonsdottir OH, Kleinman RE, Wells JC, Fewtrell MS, Hibberd PL, Gunnlaugsson G, Thorsdottir I (2014). Exclusive breastfeeding for 4 versus 6 months and growth in early childhood. Acta Paediatr.

[25] Black RE, Allen LH, Bhutta ZA, Caulfield LE, De Onis M, Ezzati M, Mathers C, Rivera J, Maternal, Group CUS (2008). Maternal and child undernutrition: global and regional exposures and health consequences. Lancet.

[26] Caulfield LE, de Onis M, Blössner M, Black RE (2004). Undernutrition as an underlying cause of child deaths associated with diarrhea, pneumonia, malaria, and measles. Am J Clin Nutr.

[27] Nandy S, Irving M, Gordon D, Smith GD. Poverty, child undernutrition and morbidity: new evidence from India. 2005. Bull World Health Organ. 2005;83(3):210–216. PMC2624218 15798845

[28] Cai X, Wardlaw T, Brown DW (2012). Global trends in exclusive breastfeeding. Int Breastfeed J.

[29] Ladomenou F, Moschandreas J, Kafatos A, Tselentis Y, Galanakis E. Protective effect of exclusive breastfeeding against infections during infancy: a prospective study. Archives of Disease in Childhood. 2010;95:1004–1008. 10.1136/adc.2009.169912 20876557

[30] Savitha M, Nandeeshwara S, Kumar MP, Raju C (2007). Modifiable risk factors for acute lower respiratory tract infections. Indian J Pediatr.

[31] Broor S, Pandey R, Ghosh M, Maitreyi R, Lodha R, Singhal T, Kabra S (2001). Risk factors for severe acute lower respiratory tract infection in under-five children. Indian Pediatr.

[32] Mihrshahi S, Ichikawa N, Shuaib M, Oddy W, Ampon R, Dibley MJ, Kabir AI, Peat JK (2003). Prevalence of exclusive breastfeeding in Bangladesh and its association with diarrhoea and acute respiratory infection: results of the multiple indicator cluster survey. J Health Popul Nutr.

[33] Lawrence RA (2011). LL: Breastfeeding: a guide for the medical profession.

[34] Chung M, Raman G, Chew P, Magula N, Trikalinos T, Lau J (2007). Breastfeeding and maternal and infant health outcomes in developed countries. Evid Technol Asses (Full Rep).

[35] Oddy WH (2001). Breastfeeding protects against illness and infection in infants and children: a review of the evidence. Breastfeeding Review.

[36] Bachrach VRG, Schwarz E, Bachrach LR (2003). Breastfeeding and the risk of hospitalization for respiratory disease in infancy: a meta-analysis. Arch Pediatr Adolesc Med.

[37] Ikem A, Nwankwoala A, Odueyungbo S, Nyavor K, Egiebor N (2002). Levels of 26 elements in infant formula from USA, UK, and Nigeria by microwave digestion and ICP–OES. Food Chem.

[38] Graf J, Meierhofer R, Wegelin M, Mosler H-J (2008). Water disinfection and hygiene behaviour in an urban slum in Kenya: impact on childhood diarrhoea and influence of beliefs. Int J Environ Health Res.

[39] Brown KH, Creed-Kanashiro H, Dewey KG (1995). Optimal complementary feeding practices to prevent childhood malnutrition in developing countries. Food Nutr Bull.

[40] Ayisi R, Wakoli A (2014). Exclusive breastfeeding practice: its implication on nutrition status, growth and morbidity pattern among infants aged 0-6 months. GJBAHS.

[41] WHO (2006). Infant and young child nutrition, Quardrnnial secretariat report.

[42] Thet MM, Khaing EE, Diamond-Smith N, Sudhinaraset M, Oo S, Aung T (2016). Barriers to exclusive breastfeeding in the Ayeyarwaddy region in Myanmar: qualitative findings from mothers, grandmothers, and husbands. Appetite.

[43] Majumder MMI, Ahmed MT, Nath SCD (2016). Evaluation of short term fever with flu like symptoms in a tertiary level Hospital in Bangladesh. Med Today.

[44] Martinez PP, King AA, Yunus M, Faruque A, Pascual M. Differential and enhanced response to climate forcing in diarrheal disease due to rotavirus across a megacity of the developing world. Proc Natl Acad Sci. 2016;113(15):4092–4097. 10.1073/pnas.1518977113 PMC4839397 27035949

[45] Boerma JT, Sommerfelt AE (1992). Demographic and health surveys (DHS): contributions and limitations. World Health Stat Q.

